# Predictors of microbial agents in dust and respiratory health in the Ecrhs

**DOI:** 10.1186/s12890-015-0042-y

**Published:** 2015-05-02

**Authors:** Christina Tischer, Jan-Paul Zock, Maria Valkonen, Gert Doekes, Stefano Guerra, Dick Heederik, Deborah Jarvis, Dan Norbäck, Mario Olivieri, Jordi Sunyer, Cecilie Svanes, Martin Täubel, Elisabeth Thiering, Giuseppe Verlato, Anne Hyvärinen, Joachim Heinrich

**Affiliations:** Institute of Epidemiology I, Helmholtz Zentrum München, Ingolstädter Landstrasse 1,German Research Centre for Environmental Health, D-85764 Neuherberg, Germany; Centre for Research in Environmental Epidemiology (CREAL), Barcelona, Spain; Universitat Pompeu Fabra (UPF), Barcelona, Spain; CIBER Epidemiología y Salud Pública (CIBERESP), Madrid, Spain; Netherlands Institute for Health Services Research (NIVEL), Utrecht, The Netherlands; Living Environment and Health Unit, National Institute for Health and Welfare, Kuopio, Finland; Institute for Risk Assessment Sciences, Division Environmental Epidemiology, Utrecht University, Utrecht, the Netherlands; Arizona Respiratory Center, University of Arizona, Tucson, AZ USA; Respiratory Epidemiology and Public Health Group, Imperial College London, London, UK; MRC-HPA Centre for Environment Health, King’s College London, London, UK; The Department of Medical Science, Occupational and Environmental Medicine, Uppsala University, Uppsala, Sweden; Department of Occupational Medicine, University of Verona, Verona, Italy; IMIM (Hospital del Mar Medical Research Institute), Barcelona, Spain; Department of Occupational Medicine, Centre for International Health, University of Bergen and , Haukeland University Hospital, Bergen, Norway; Division of Metabolic Diseases and Nutritional Medicine, Dr von Hauner Children’s Hospital, Ludwig-Maximilians-University, Munich, Germany; Epidemiology and Medical Statistics, University of Verona, Verona, Italy

**Keywords:** Molds, Fungi, Microbials, Indoor Air, Asthma, Airways

## Abstract

**Background:**

Dampness and mould exposure have been repeatedly associated with respiratory health. However, less is known about the specific agents provoking or arresting health effects in adult populations. We aimed to assess predictors of microbial agents in mattress dust throughout Europe and to investigate associations between microbial exposures, home characteristics and respiratory health.

**Methods:**

Seven different fungal and bacterial parameters were assessed in mattress dust from 956 adult ECRHS II participants in addition to interview based home characteristics. Associations between microbial parameters and the asthma score and lung function were examined using mixed negative binomial regression and linear mixed models, respectively.

**Results:**

Indoor dampness and pet keeping were significant predictors for higher microbial agent concentrations in mattress dust. Current mould and condensation in the bedroom were significantly associated with lung function decline and current mould at home was positively associated with the asthma score. Higher concentrations of muramic acid were associated with higher mean ratios of the asthma score (aMR 1.37, 95%CI 1.17-1.61). There was no evidence for any association between fungal and bacterial components and lung function.

**Conclusion:**

Indoor dampness was associated with microbial levels in mattress dust which in turn was positively associated with asthma symptoms.

**Electronic supplementary material:**

The online version of this article (doi:10.1186/s12890-015-0042-y) contains supplementary material, which is available to authorized users.

## Background

“Bio-aerosols’, such as bacteria, fungi, pollen, viruses and their by-products, as well as fragments from living organisms, such as allergens, occur ubiquitously in communities and most of the time we are living in peaceful co-existence [1,2]. Home and personal characteristics can substantially influence the microbial profile in indoor environments [3,4]. In two recent publications of the European Community Respiratory Health Survey (ECRHS), exposure to dampness and mould at home was significantly associated with the onset of asthma [5] and lung function decline in women [6]. Apart from that, another previous ECRHS study among the same study population demonstrated that the presence of dogs and cats within the home environment was significantly associated with higher mattress dust concentrations of endotoxin, a bacterial lipopolysaccharide (LPS) which can be found in the outer membrane of gram-negative bacteria [7]. Pet keeping has been associated with heterogeneous effects in relation to atopy in children and adult populations [8–13]. In addition, tobacco smoke indoors has been also selected as a further potential predictor for microbial agents concentration [7,14] and smoking is in turn also positively associated with respiratory and asthmatic symptoms [15].

As far as we are aware, studies on the exposure to multiple microbial agents in the home environment in adult populations are rare, particularly at larger sample sizes, covering a wide geographical area. In general, describing the ‘microbial world’ in indoor environments is complicated by the variability and different sources of microbial components as well as the lack of standardized exposure assessment and analyses methods [16]. In the past, culture-based methods have been used to measure fungal and bacterial exposure. However, these methods are dependent on viability of the sampled material in combination with the overgrowing of some specific species [2]. Recently, molecular methods, such as quantitative polymerase chain reaction (qPCR) were introduced estimating the microbial exposure indoors independent from viability. The targets to be detected can be species, genera or more broadly defined taxa, which allows for more detailed information on the exposure [1].

In the follow-up phase of the ECRHS II, microbial agents in mattress dust samples collected in 22 study centres from 10 European countries were determined with molecular and chemical analysis. We took advantage of this unique frame-work of the ECRHS II to determine whether selected home characteristics such as mould and dampness (observed and self-reported), pet keeping, current smoking and the location of the study centre in Europe are suitable predictors of bacterial and fungal biomarkers. Second, we aimed to explore whether these selected home characteristics in addition to quantitatively measured exposure to the microbial agents in mattress dust are associated with current asthma symptoms and lung function in adults. This study was performed as part of the European-based HITEA study on indoor air in relation to the development of asthma and allergies.

## Methods

### Study population and mattress dust sample selection

The European Community Respiratory Health Survey (ECRHS) is a multicenter cross-sectional study (1991–1993) covering a large geographical area in Europe [17]. In 1999–2001 (ECRHS II), the participants were followed up in 29 centers in a random population sample of adults aged 20–44 years at the baseline survey (ECRHS). Twenty-two centers from 10 European countries agreed to take part in a detailed assessment of home exposures, including mattress dust samples [18–20]. The current investigation is part of the “Health effects of indoor pollutants: Integrating microbial, toxicological and epidemiological approaches (HITEA) study”. For this project, at total of 999 mattress dust samples were randomly selected from the ECRHS II random sample of all 22 participating study centers which took part in the detailed indoor assessment [7,18]. Priority was given to those participants who had not moved home between 1992–1994 and 2000–2002 (624/955, 64%) who provided a blood sample for serum specific Immunoglobulin E (IgE) testing. The countries and study centres included are Iceland (Reykjavik), Sweden (Uppsala, Umea, Gothenburg), Estonia (Tartu), Germany (Hamburg, Erfurt), UK (Norwich, Ipswich), Belgium (Antwerp Centre, Antwerp South), France (Paris, Grenoble), Switzerland (Basel), Italy (Verona, Pavia, Turin), and Spain (Oviedo, Galdakao, Barcelona, Albacete, Huelva).

### Selection and analysis of microbial agents

In the central laboratory in Imperial College, UK, the dust samples were sieved (1 mm) to remove larger particles and to homogenise the dust for extraction [20] and stored. In 2008, the dust was split into homogeneous fractions of 50–70 mg, and transferred to pre-weighed 10 mL polystyrene vials with screw caps (Sterilin, Newport, UK). One vial was sent within days to Utrecht University (The Netherlands) and extracted for bacterial endotoxin and (1,3)-ß-D-glucan [7]. Another vial was delivered to THL in Kuopio (Finland) for fungal and bacterial qPCR analysis and for the analysis of muramic acid.

The microbial agents analysed were selected for being promising targets – i.e. showing positive or negative associations with respiratory health parameters in a previous investigation using a larger set of molecular markers in a sub-sample of 400 individuals of the ECRHS2 (manuscript under preparation). DNA was extracted from 20 mg (17.9-20.8 mg, 5th-95th percentile) of accurately weighed dust using bead beating for mechanical cell disruption as described [21] with the modification of the KingFisher DNA extraction robot (Thermo Scientific) and NucleoMag plant kit (Macherey-Nagel). The qPCR laboratory analyses and calculations were performed as described by Kaarakainen and colleagues [22] using the ABI Prism 7000 (Applied Biosystems) and the RotorGene 3000 (Corbett Life Science) equipment. Muramic acid analyses were performed with separate dust aliquots of 8–10 mg as described by Kärkkäinen and colleagues [21]. Overall, 956 samples were analysed for muramic acid, qPCR analyses were performed for *Cladosporium herbarum*, the group *Penicillium spp., Aspergillus spp. and Paecilomyces variotii* [23] (Haugland & Vesper, US pat. 2002; 6 387 652), *Mycobacterium spp.* [24], and gram-positive as well as gram-negative bacteria [21].

In addition there was also information available on (1,3)-ß-D-glucan and endotoxin in a subset of the study participants (N = 783). (1,3)-ß-D-glucans were measured with the inhibition enzyme immunoassay described by Douwes et al. [25] using the insoluble ECRHS house dust residues stored at −20°C after sequential extraction of endotoxins (in water-Tween) and protein/carbohydrate allergens (in PBS-Tween), both at room temperature. (1,3)-ß-D-glucans in the residues were solubilised by extraction at 120°C in PBS-Tween [26]. The (1,3)-ß-D-glucan inhibition EIA makes use of affinity-purified polyclonal rabbit antibodies, and with the algae-derived glucan laminarin as the coated antigen and the calibration standard, as described previously [25]. For the current investigation, only the results of the (1,3)-ß-D-glucan measurements will be considered in relation to health, as results on endotoxin have been published previously [19]. However, endotoxin will be included, investigating the correlation between different microbial parameters.

### Home and personal characteristics

During home inspection, a face-to-face interview was conducted with each participant by trained inspectors (‘indoor protocol’). For the current investigation we focused on signs of dampness and mould, exposure to pets, current smoking and whether the bed is placed in the living room as those characteristics have been associated with microbial levels as well as with asthma and respiratory health in previous studies [5–7]. From the indoor protocol, inspector observed information on current dampness and mould problems in the bedroom as well as self-reported information during inspection on condensation on bedroom windows in the morning during winter was used. From the main questionnaire, we included self-reported information on persistent (current and ever) and ever water damage in the building, persistent (current and ever) and ever damp spots inside the home as well as persistent (current and ever) and ever mould or mildew inside the home. In addition, having a cat or dog and allowance to enter the bedroom, current smoking status and whether the bed is placed in the living room was also obtained from the main questionnaire. To explore whether there is a geographical gradient influencing the microbial agent levels in mattress dust, information on the latitude regarding the study centers was also included. A detailed description of the questions on the selected home characteristics can be found in the Additional file 1.

### Current asthma symptoms and lung function parameters

The asthma score [27] was used as semi-quantitative measure of questionnaire based asthma symptoms in the past 12 months. The ordinal score, which ranges from 0 to 5 counts positive responses regarding the following: wheeze with breathlessness, chest tightness, attacks of shortness of breath (SOB) at rest, SOB after exercise, and being woken by SOB. We also considered the results of lung function measurements in which each participant had been given up to nine attempts to provide two technically satisfactory forced expiratory manoeuvres. The highest recorded FEV_1_ and FVC, which fulfilled the American Thoracic Society criterion for reproducibility, were used as the outcomes [6,19]. In addition, blood samples had been analysed for specific IgE. Allergic sensitization to aero-allergens was defined as the presence of >0.35 kU/l of specific IgE to house dust mite, cat, timothy grass and/or *Cladosporium herbarum*.

### Statistical analysis

Concentrations of fungal and bacterial agents were naturally log-transformed. Correlations between all microbial agents (log-transformed) were assessed using the Pearson product–moment correlation coefficient (r). The associations between the selected home characteristics and microbial parameters in mattress dust were assessed using linear mixed models, using random effects.

Effect estimates are presented per interquartile range (IQR) increase in log-linearly associated microbial exposure.

The linearity of the associations between exposure to microbial agents and health was tested with generalized additive mixed models (GAMMs). The association between exposure to concentrations of microbial agents (log-transformed) and exposure to selected home and personal characteristics with the asthma score was assessed using mixed negative binomial regression analysis [28]. The results are presented as adjusted mean ratios (aMR) with corresponding 95% confidence intervals (95% CI). Exposure to microbial agents in mattress dust and selected home and personal characteristics in relation to weight and height adjusted lung function parameters FEV_1_ and FVC was evaluated using linear mixed models. The results are presented as adjusted estimates (ß) with corresponding 95% confidence intervals (95% CI). For all association analyses, mixed models with random intercept were used. Confounders were selected based on previous literature and association analyses (*X*^*2*^ test, Kruskall Wallis) with the asthma score and lung function parameters, respectively (p < 0.1). The asthma score model was adjusted for sex, damp patches in the bedroom, condensation on bedroom windows, bed placed in living room, current smoking and season of dust sampling. The lung function model (FEV_1_ and FVC) was adjusted for sex, education, height, weight, damp patches in the bedroom, current water damage, bed placed in living room, current smoking and season of dust sampling. For the association analyses between exposure to selected home and personal characteristics in relation to respiratory outcomes, the exposure to measured microbial agents was additionally considered in case there was a significant effect with the health outcomes in the respective models. All statistical analyses were performed using the statistical software R, version 2.14.1 (www.r-project.org).

### Ethical approval

Written informed consent was obtained from all participants and approved by the local ethics committees in each region: Reykjavik, Island (The National Bioethics Committee, Reykjavík, Island), Umea, Uppsala, Gothenburg, Sweden (Regional Ethical Committee in Uppsala, Sweden), Erfurt, Hamburg, Germany (Ethic Committee of the Bavarian State Chamber of Physicians, Germany), Norwich, UK (Norwich District Ethics Committee), Ipswhich, UK (Ipswich- East Suffolk Local Research Ethics Committee), Antwerp S, Antwerp C, Belgium (Adviescommissie Medische Ethiek UZA-UA (CME), Universitair Ziekenhuis Antwerpen (UZA)), Paris, Grenoble, France (Ethics committee Paris Bichat-Claude Bernard), Turin, Italy (Comitato Etico dell’Azienda Sanitaria Locale TO/2 di Torino), Verona, Italy (Comitato Etico per la Sperimentazione dell’Azienda Ospedaliera Istituti Ospitalieri di Verona), Pavia, Italy (Comitato di Bioetica della Fondazione IRCCS Policlinico San Matteo di Pavia), Barcelona, Spain (Comité Ético de Investigación Clínica del Instituto Municipal de Asistencia Sanitaria, Barcelona, Spain), Albacete, Spain (Comité de Ética e Investigación de Complejo Hospitalario de Albacete, Spain), Huelva, Spain (Comisión de Investigación del Hospital Juan Ramón Jiménez de Huelva, Spain), Oviedo, Spain (Comité Ético de Investigación Clínica Regional, Hospital Universitario Central de Asturias, Oviedo, Spain), Galdakao, Spain (Comité Ético de Investigación del Hospital de Galdakao, Spain), Tartu, Estland (Research Ethics Committee of the University of Tartu, Estland), Basel, Switzerland (Swiss Academy of Medical Sciences and the ethics committee of Basel).

## Results

### Study population and home characteristics

Study population as well as selected home and personal characteristics are given in Table 1. In total, 956 participants were on average 44 years old and finished their education at 20 years of age. The majority of the study population reported no asthma symptoms in the past year (64%) and only 2% had a score of more than 3 symptoms. The medians of FEV_1_ and FVC were 3470 and 4230 millilitre, respectively.

**Table 1:** **Characteristics of the study population and the home environment**

	**n/N (%)**
**Females**	486/956 (51%)
**Age** (in years, median)	44 years
**Education** (age, median)	at 20 years
**Asthma Score, past 12 months**	
0 (no symptoms)	603/949 (64%)
1	208/949 (22%)
2	95/949 (10%)
3	20/949 (2%)
4	14/949 (2%)
5	9/949 (1%)
**FEV**_**1**_ (in milliliter, 1st second, median)	3470 ml/s
**FVC** (in milliliter, median)	4230 ml
**Observed dampness and mould in the bedroom**	
Damp patches on the walls/ceilings in the bedroom	71/904 (8%)
Mould or mildew on the walls/ceilings in the bedroom	47/904 (5%)
Reported condensation on the bedroom windows (winter)	208/899 (23%)
**Self-reported signs of dampness and mould at home**	
Wet or damp spots in the last 12 months	197/950 (21%)
Mould in the home	
Current	150/950 (16%)
Ever before	72/950 (8%)
Water damage in the home	
Current	94/930 (10%)
Ever before	189/930 (20%)
**Exposure to tobacco smoke**	
Current smoking	299/954 (31%)
Former smoking	253/954 (27%)
Never smoked	402/954 (42%)
**Cat in the home**	
Cat in the home	182/955 (19%)
Cat in the home **and** allowed in the bedroom	132/955 (14%)
**Dog in the home**	
Dog in the home	156/955 (16%)
Dog in the home **and** allowed in the bedroom	82/955 (9%)
**Season of dust sampling**	
Winter	227/907 (25%)
Spring	256/907 (28%)
Summer	129/209 (14%)
Autumn	295/907 (33%)
**Bed in the living room**	54/955 (6%)

Reported condensation on the bedroom windows during inspection was quite common (23%). Inspector-observed damp spots and signs of mould in the bedroom were reported with 8% and 5%, respectively. Current self-reported wet or damp spots in the home, current mould and current water damage were found to be more prevalent with 21%, 16% and 10%, respectively. Ever having mould inside the home and ever having water damage to the building or its contents was reported with 8% and 20%, respectively. Nearly one third of the study population were current smokers (31%). Cat ownership was slightly more common (19%) than dog ownership (16%) and a considerable portion of both animals were allowed to be in the bedroom (14% and 9%, respectively). Dust sampling was not equally distributed among the seasons; whereas almost an equivalent amount of dust samples was taken during winter and spring (25% and 28%, respectively), less dust sampling was performed in the summer season (14%) and more during autumn (33%). Lastly, 6% of the study participants sleep in the living-room.

### Distribution and correlation of microbial compounds and species throughout Europe

**Figure 1:**
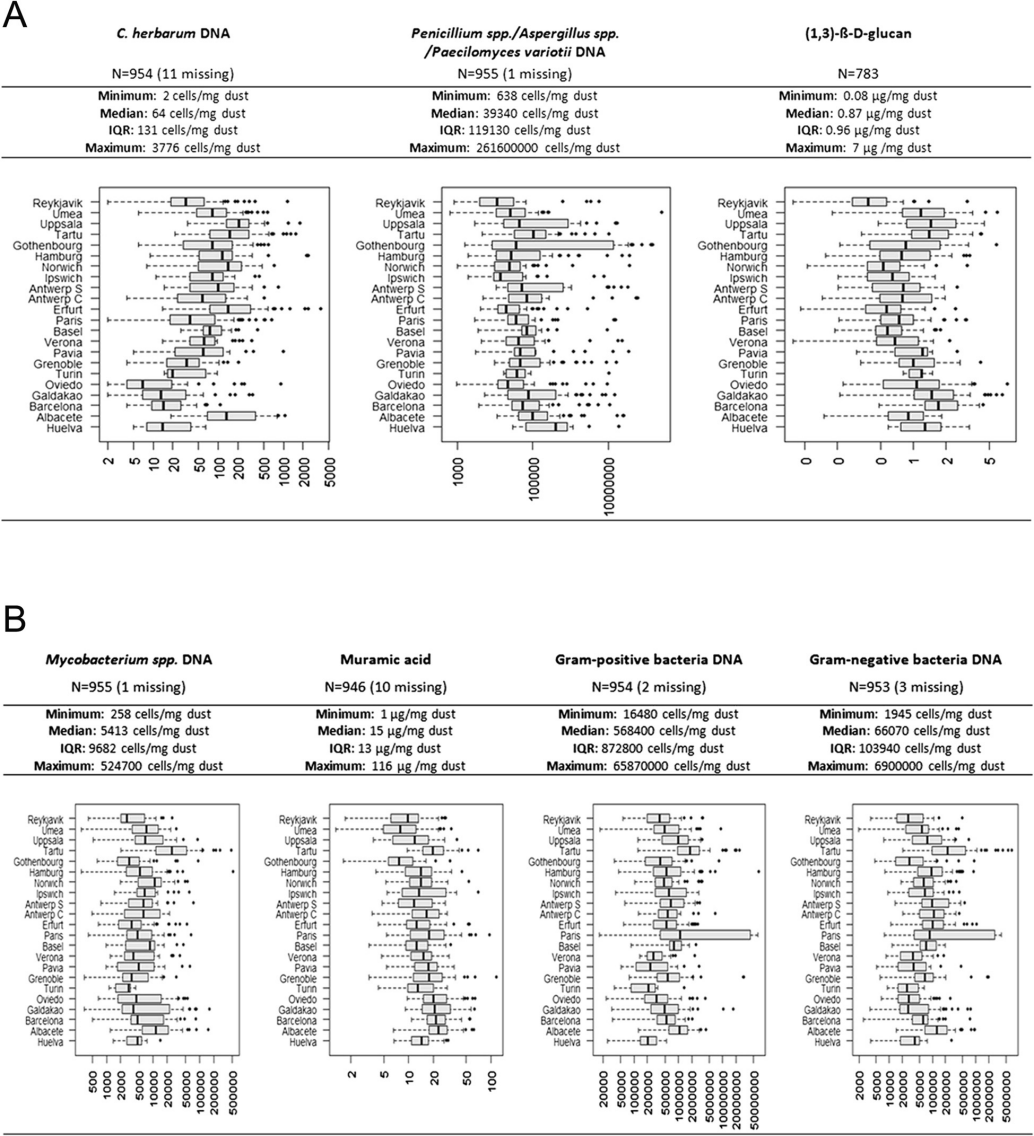
Distribution of the (log-transformed) microbial agents measured from mattress dust samples. The boxplots are stratified by study center and ordered by latitude. The x-axis shows the range of the concentrations in original scale. **A.** Fungal agents **B.** Bacterial agents.

Of 999 selected dust samples, 956 were analysed for microbial parameter and additional information on (1,3)-ß-D-glucan was available in a subset of 783 participants. The distribution and geographical variation of the microbial agent levels, stratified by study centre is displayed by boxplots in Figures 1A and B. The summary statistics yielded that the total median (original scales) was 64 cells per mg dust for *Cladosporium herbarum* DNA, 39340 cells/mg for *Penicillium* spp./*Aspergillus* spp./*Paecilomyces variotii* group DNA, 0.87 μg/mg for (1,3)-ß-D-glucan, 5413 cell/mg for *Mycobacterium spp.* DNA, 15 μg/mg for Muramic acid, 568400 cells/mg for gram-positive and 66070 cells/mg for gram-negative bacteria. There was considerable variation between the European centres, except for muramic acid levels.

Except for C. herbarum and muramic acid, as well as for (1,3)-ß-D-glucan and gram-positive bacteria for which no correlation has been observed, the remaining measured microbial agents in mattress dust were significantly correlated with each other as shown in Table 2. However, higher correlations (above 0.50) were only observed for *Mycobacterium spp.* with gram-negative bacteria (Pearsons r = 0.55) and between gram-positive and gram-negative bacteria (Pearson’s r = 0.82).

**Table 2:** **Correlation between the microbial agent concentrations in mattress dust (Pearson’s r, log-transformed data)**

	***C. herbarum*** **DNA**	***Pen./Asp./Paec.*** **DNA**	**(1,3)-ß-D-glucan**	***Mycobacterium spp.*** **DNA**	**Muramic acid**	**Gram-pos. bacteria DNA**	**Gram-neg. bacteria DNA**	**Endotoxin**
***C. herbarum*** **DNA**								
***Pen./Asp./Paec.*** **DNA**	0.14***							
**(1,3)-ß-D-glucan**	0.12***	0.35***						
***Mycobacterium spp.*** **DNA**	0.49***	0.28***	0.25***					
**Muramic acid**	**0**	0.17***	0.22***	0.25***				
**Gram-pos. bacteria DNA**	0.32***	0.24***	**0**	0.45***	0.43***			
**Gram-neg. bacteria DNA**	0.45***	0.28***	0.12***	0.55***	0.31***	0.82***		

*p < 0.05, **p < 0.01, ***p < 0.001.

### Predictors for levels of microbial agents in mattress dust

In Table 3, associations in mutual adjusted models between microbial agent concentrations in mattress dust and selected home, personal and environmental characteristics are shown. Mattress dust from homes of cat and dog owners had on average higher levels of microbial components compared to homes without pets, while the associations with dog ownership were stronger and observed for all microbial parameters measured. From the fungal compounds measured, *Penicillium* spp./*Aspergillus* spp./*Paecilomyces variotii* group was associated with signs of mould and dampness at home. Reported condensation during inspection on the bedroom windows and self-reported damp spots in the home significantly increased the levels of *Penicillium* spp./*Aspergillus* spp./*Paecilomyces variotii* DNA in mattress dust. Observed signs of dampness in the bedroom were associated with higher concentrations of DNA from gram-positive as well as gram-negative bacteria compared to non-damp bedrooms. Previous water damage somewhere in the home environment was significantly associated with a higher mean ratio of muramic acid. Current smoking significantly increased the levels of muramic acid and DNA from gram-positive bacteria. With increasing latitude, there were significantly higher concentrations of DNA from *Cladosporium herbarum* and significantly lower concentrations of DNA from *Penicillium* spp./*Aspergillus* spp./*Paecilomyces variotii* and muramic acid in mattress dust. Dust samples taken in summer and autumn were associated with higher concentrations of *Cladosporium herbarum* and (1,3)-ß-D-glucan. Dust samples taken from spring to autumn were associated with lower concentrations of bacterial compounds in mattress dust, significantly for muramic acid in spring and gram-positive bacteria in autumn.

**Table 3:** **Associations in mutual adjusted models between fungi and bacterial agent concentrations in mattress dust and selected home**

**Home, personal and environmental characteristics**							
**Characteristics**	***C. herbarum*** **DNA MR (95% CI)**	***Pen./Asp./Paec.*** **DNA MR (95% CI)**	**(1,3)-ß-D-glucan MR (95% CI)**	***Mycobac. spp.*** **DNA MR (95% CI)**	**Muramic acid DNA MR (95% CI)**	**Gram-pos bac DNA MR (95% CI)**	**Gram-neg bacDNA MR (95% CI)**
**Damp patches in the bedroom** (observed)	1.19 (0.79-1.80)	1.11 (0.50-2.48)	1.02 (0.82-1.28)	1.39 (0.90-2.14)	1.19 (0.96-1.49)	**1.87 (1.16-3.00)**	**1.70 (1.09-2.67)**
**Visible mould in the bedroom**(observed)	1.06 (0.66-1.69)	0.95 (0.38-2.37)	1.23 (0.94-1.59)	0.87 (0.53-1.43)	0.98 (0.76-1.26)	0.77 (0.45-1.32)	0.77 (0.46-1.28)
**Condensation in bedroom** (reported during inspection)	0.94 (0.78-1.13)	**1.87 (1.31-2.67)**	1.10 (0.99-1.23)	0.92 (0.76-1.12)	0.91 (0.83-1.01)	0.91 (0.73-1.12)	1.03 (0.84-1.26)
**Wet or damp spots in the last 12 months**	1.13 (0.92-1.40)	**1.67 (1.11-2.51)**	1.09 (0.96-1.23)	1.15 (0.92-1.44)	0.93 (0.83-1.05)	0.80 (0.63-1.02)	1.07 (0.85-1.34)
**Mould in the home:** Current	0.99 (0.78-1.24)	1.26 (0.81-1.97)	0.93 (0.82-1.06)	0.92 (0.72-1.18)	0.98 (0.86-1.11)	0.99 (0.76-1.29)	1.03 (0.80-1.32)
Ever before	0.88 (0.66-1.16)	0.86 (0.50-1.50)	1.06 (0.90-1.26)	0.91 (0.67-1.22)	1.14 (0.98-1.34)	1.05 (0.75-1.45)	1.06 (0.77-1.44)
**Water damage in the home:** Current	1.07 (0.83-1.39)	0.85 (0.51-1.41)	1.00 (0.86-1.17)	0.95 (0.73-1.25)	1.03 (0.90-1.18)	1.27 (0.94-1.71)	1.14 (0.86-1.51)
Ever before	0.94 (0.77-1.14)	0.85 (0.59-1.24)	0.97 (0.87-1.08)	0.89 (0.72-1.09)	**1.15 (1.04-1.28)**	0.88 (0.71-1.10)	0.88 (0.71-1.08)
**Cat in the home**	**1.52 (1.09-2.12)**	1.14 (0.60-2.16)	1.10 (0.91-1.32)	1.32 (0.94-1.87)	1.13 (0.94-1.35)	1.26 (0.86-1.84)	1.08 (0.76-1.56)
**Cat also allowed in bedroom**	**1.47 (1.18-1.83)**	0.91 (0.59-1.39)	**1.14 (1.01-1.29)**	**1.46 (1.16-1.84)**	1.12 (0.99-1.26)	1.17 (0.91-1.51)	1.25 (0.98-1.59)
**Dog in the home**	**1.61 (1.22-2.14)**	**2.50 (1.44-4.31)**	**1.36 (1.16-1.61)**	**1.79 (1.33-2.41)**	1.15 (0.99-1.34)	1.33 (0.96-1.83)	**1.65 (1.21-2.24)**
**Dog also allowed in bedroom**	**2.31 (1.74-3.05)**	1.34 (0.77-2.32)	**1.24 (1.06-1.45)**	**2.23 (1.66-3.01)**	**1.28 (1.09-1.49)**	**2.06 (1.49-2.85)**	**2.20 (1.61-3.01)**
**Current smoking**	1.10 (0.92-1.31)	0.88 (0.63-1.25)	1.00 (0.91-1.11)	0.92 (0.76-1.10)	**1.14 (1.03-1.25)**	**1.32 (1.08-1.62)**	1.10 (0.90-1.33)
**Bed in living room**	0.53 (0.07-3.82)	1.53 (0.32-7.34)	1.64 (0.68-3.93)	0.30 (0.04-2.43)	1.09 (0.37-3.19)	0.42 (0.04-4.13)	0.25 (0.03-2.23)
**Latitude (per 10°N)**	**1.63 (1.05-2.54)**	**0.56 (0.40-0.79)**	0.87 (0.69-1.10)	1.06 (0.80-1.41)	**0.74 (0.64-0.84)**	1.22 (0.85-1.76)	1.15 (0.78-1.68)
Spring**Season** (ref. winter)	0.83 (0.67-1.02)	0.83 (0.55-1.24)	0.92 (0.82-1.04)	0.89 (0.71-1.10)	**0.89 (0.79-0.99)**	0.81 (0.64-1.04)	0.80 (0.64-1.01)
Summer	**1.80 (1.40-2.31)**	0.80 (0.49-1.29)	**1.22 (1.06-1.41)**	1.12 (0.86-1.46)	1.04 (0.90-1.19)	0.82 (0.62-1.10)	0.84 (0.64-1.11)
Autumn	**1.84 (1.50-2.25)**	0.82 (0.55-1.21)	**1.16 (1.03-1.30)**	1.01 (0.82-1.25)	0.99 (0.88-1.10)	**0.71 (0.56-0.89)**	0.83 (0.67-1.04)

**Personal and environmental characteristics**. Adjusted Mean Ratio (MR) and 95% Confidence **Interval (95% CI) per interquartile range (IQR) increase**. Numbers in bold indicate significant associations.

### Association between selected home and personal characteristics with current asthma symptoms and lung function parameters

Having a damp or mouldy home or bedroom was associated with higher adjusted mean ratios of the asthma score, significantly for reported current exposure to mould at home (aMR 95%CI: 1.41 (1.07-1.85)) as shown in Table 4. There was a significant decline of FEV_1_ with reported condensation on bedroom windows (aß 95%CI: −90 (−160; −10)) and for FVC with observed visible mould in the bedroom (aß 95%CI: −140 (−280; −3)). Current smoking was associated with a significantly increased risk of current asthma symptoms (aMR 95%CI: 1.85 (1.44-2.36)) and borderline significantly associated with reduced FEV_1_ (aß 95%CI: −70 (−140; 1)). Sleeping in the living room was significantly associated with higher adjusted mean ratios of the asthma score (aMR 95%CI: 1.63 (1.10-2.42)) as well as lung function decline in FEV_1_ (aß 95%CI: −230 (−390; −80)) and FVC (aß 95%CI: −210 (−410; −10)). Cat owners showed significantly improved FEV_1_ values (aß 95%CI: 140 (10; 280)) compared to participants without cats at home. Overall, there was no major change in the effect estimates after additional adjusting for IQR increase of muramic acid in mattress dust, however, the confidence intervals were wider (data not shown).

**Table 4:** **Association between exposure to IQR increase of microbial agent concentration in mattress dust and selected home and personal characteristics with the asthma score and lung function parameters FEV**_**1**_ **and FVC (mL)**

		**Asthma score** ^*^ **aMR (95% CI)**	**FEV**_**1**_^**$**^ **aß (95% CI)**	**FVC** ^**$**^ **aß (95% CI)**
**Microbial Agents per IQR**	***C. herbarum*** **DNA**	0.92 (0.78-1.09)	20 (-30; 80)	20 (-50; 80)
	***Pen. /Asp./Paec. Variotii*** **DNA**	1.07 (0.94-1.21)	-10 (-50; 30)	10 (-30; 60)
	**(1,3)-ß-D-glucan**	0.94 (0.81-1.10)	-10 (-100-50)	-40 (-110;30)
	***Mycobacterium spp.*** **DNA**	1.08 (0.93-1.26)	10 (-40; 50)	-20 (-80; 30)
	**Muramic acid**	**1.37 (1.17-1.61)**	3 (-50; 50)	0.3 (-60; 60)
	**Gram-positive bacteria DNA**	1.07 (0.94-1.21)	-4 (-40;30)	-20 (-60; 30)
	**Gram-negative bacteria DNA**	1.04 (0.91-1.20)	-10 (-50; 30)	-20 (-70; 30)
**Dampness and Mould**	**Damp patches in the bedroom**	1.40 (0.97-2.01)	-120 (-240; 2)	-120 (-240; 2)
	**Visible mould in the bedroom**	1.32 (0.84-2.07)	-100 (-240; 40)	**-140 (-280; -3)**
	**Condensation on bedroom windows**	1.22 (0.96-1.57)	**-90 (-160; -10)**	-70 (-170; 20)
	**Dampness at home past 12 months**	1.24 (0.97-1.59)	4 (-80; 90)	50 (-50; 150)
	**Mould at home**			
	**Current**	**1.41 (1.07-1.85)**	-10 (-100; 80)	60 (-50; 170)
	**Ever**	1.12 (0.75-1.66)	-70 (-190; 50)	-60 (-200; 80)
	**Water damage**			
	**Current**	1.10 (0.77-1.56)	-10 (-120; 90)	-10 (-140; 120)
	**Ever**	1.16 (0.90-1.51)	-10 (-90; 70)	-20 (-120; 80)
**Home / Personal**	**Cat in the home**	1.04 (0.66-1.66)	**140 (10; 280)**	160 (-10; 320)
	**Cat in bedroom**	0.93 (0.68-1.27)	-3 (-100; 90)	-10 (-130; 100)
	**Dog at home**	1.12 (0.76-1.63)	-10(-130; 100)	-60 (-200; 80)
	**Dog in bedroom**	1.05 (0.72-1.53)	50 (-60; 160)	40 (-100; 180)
	**Current smoking**	**1.85 (1.44-2.36)**	-70 (-140; 1)	0.1 (-90; 90)
	**Bed in living room**	**1.63 (1.10-2.42)**	**-230 (-390; -80)**	**-210 (-410; -10)**

Results are displayed as adjusted Mean Ratios (aMR) and adjusted ß (aß) with 95% confidence intervals (95% CI), respectively.

*Model asthma score adjusted for: sex, damp patches in the bedroom, condensation on bedroom windows, bed is in living room, current smoking, and season of dust sampling.^$^Model lung function adjusted for: sex, education, height, weight, damp patches in the bedroom, current water damage in the building, bed is in living room, current smoking and season of dust sampling. Numbers in bold indicate significant associations.

### Association between microbial agents in mattress dust with current asthma symptoms and lung function parameters

In Table 4, the combined adjusted MRs (aMR) for the associations between exposure to fungal and bacterial components in mattress dust with current asthma symptoms (‘asthma score’) and lung function parameters are shown. All associations between the exposure to higher concentrations and the selected health outcomes showed a linear relationship. Exposure to higher levels of muramic acid was significantly positively associated with the asthma score (aMR 95%CI: 1.37 (1.17-1.61). No statistically significant associations were seen for exposure to the remaining fungal and bacterial components in relation to the asthma score. Overall, there was also no evidence of an association of FEV_1_ and FVC with exposure to microbial agent levels.

## Discussion

In this multinational epidemiological cohort study we observed in mutual adjusted analysis that current signs of dampness in the bedroom or home environment and having a cat or dog were significant predictors for higher fungal and bacterial agent concentrations in mattress dust. We further observed that higher concentrations of muramic acid levels in mattress dust were significantly associated with current asthma symptoms. Questionnaire based signs of current mould and condensation in the bedroom were significantly associated with lung function decline. Current mould in the home environment was significantly associated with increased adjusted mean ratios of the asthma score. Harmful effects of current smoking and sleeping in the living room were observed with both, current asthma symptoms and lung function decline. In contrast, cat ownership was associated with a significant improvement of FEV_1_. No association was observed with exposure to the remaining measured bacterial and fungal compounds and there was no evidence of an association with lung function parameters FEV_1_ and FVC.

Numerous studies have analysed the relationship between living in a damp and mouldy environment and effects on respiratory health in children and adult populations. In a recent ECRHS investigation, Norbäck and colleagues observed that dampness and mould at home contribute to asthma incidence in adults [5]. The same authors also reported a decline in lung function (FEV_1_) among those subjects with problems of dampness and indoor mould growth in the home [6]. Further, the RHINE study, a follow-up of the ECRHS I in northern European countries found that dampness at home was associated with the onset of respiratory symptoms and a less common remission of nocturnal respiratory symptoms among the subjects [29]. Yet, the actual causal agents related to signs of dampness and mould at home and suggested to be responsible for the observed health effects are not known. Within our study, we tried to disentangle the effects of exposure to selected home characteristics and quantitatively measured microbial agents on respiratory outcomes. As expected, we observed harmful effects of current exposure to a damp and mouldy home environment on asthma symptoms and lung function. We further observed that exposure to higher concentrations of bacteria derived muramic acid was also associated with an increased risk of current asthma symptoms. In turn, we could also determine that previous water damage somewhere in the home significantly resulted in a higher mean ratio of muramic acid in mattress dust. Further, current damp patches in the bedroom were also associated with a (non-significant) increase of muramic acid concentrations.

Muramic acid is a cell wall component, predominantly derived from gram-positive bacteria and it is recognized by the innate and humoral immune system by microorganism-associated molecular patterns (MAMPs) [30,31]. Until now, only little is known about the medical consequences of exposure to measured bacterial agents in settled dust from indoor environments in adult populations. One study in Russian and Finnish Karelia compared pooled house dust samples and observed up to 20-fold higher contents of muramic acid in Russian homes. The ecological study hypothesized, that the different composition of bacterial profiles in the house dust samples might be responsible for the lower occurrence of atopy and atopic diseases in children in Russian Karelia compared to Finnish Karelia [32]. In other epidemiological studies, endotoxin, a component of the bacterial cell wall of mostly gram-negative bacteria [33] has been widely used as a proxy for bacterial exposure in settled dust. In a previous ECRHS investigation among the same study participants, there was no evidence of an association between exposure to endotoxin levels in mattress dust and the asthma score, however, an association with lung function might be modified by CD14 genotype [19]. A nationwide study of more than 2500 adult subjects in the U.S. (the National Survey of Allergens in Housing (NSLAH)), reported that increased exposure to endotoxin from bedding was most strongly associated with wheezing ever, wheezing in the past month and within the past year in adjusted analysis. Increased, but not statistically significant associations were also found for asthma symptoms in the past 12 months as well as current asthma medication use [34]. In addition, in another investigation among the same subjects, an increased concentration of the *Alternaria alternata* antigen in living-room floor dust was associated with current asthma in adjusted analysis whereas there was no effect observed for exposure to the fungal species sampled from bedding [35]. Within our study, we also observed higher levels of DNA from *Penicillium spp./Aspergillus spp./Paecilomyces variotii* and DNA from gram-positive and gram-negative bacteria in mattress dust from homes with signs of mould and dampness. Although it is known from literature that *Penicillium spp., Aspergillus spp. and Paecilomyces variotii* are associated in the context of dampness or mould [1,36,37], we could not determine any effect of exposure to higher concentrations in mattress dust in relation to respiratory health outcomes. To a good extent, the amount of gram-positive bacteria, but also gram-negative bacteria in mattress dust can be attributed to human sources, especially in mattress dust [38]. The importance of the human itself as a source for microbial agents (‘microbiome’) in relation to asthma and allergic health is still an under investigated issue but with increasing importance and research interest [39].

Our study underscores the importance of exposure to bacteria compounds next to those from fungal origin in relation to respiratory health. Further, compared to studies in occupational and farming environments where high loads and concentrations of airborne fungal and bacterial agents were associated with respiratory symptoms [33,40,41], our study provided insights on the consequences of moderate measured fungal and bacterial concentrations in home environments. In general, the strengths of this study are the moderate sample size, covering a large European area with standardized exposure assessment and a standardized measurement of microbial agents in a central laboratory. One general limitation might be that it is not possible to assign the effect of muramic acid on asthma symptoms to bacteria species. Also, whereas an effect was observed with exposure to muramic acid and current asthma symptoms, there was no evidence of an association with lung function parameter FEV_1_ and FVC. We investigated whether this finding can be attributed to atopy, expressed by specific sensitization to Immunoglobulin E to inhalant allergens. However, no significant association could be observed when investigating atopic and non-atopic subjects in stratified analyses (data not shown). Further, we observed a significant improvement of FEV_1_ lung function parameters in study participants owning a cat, however, there was no improvement for cat owners who allowed their cat to be in the bedroom. The latter indicates that this result might be influenced by a selective cat avoidance as it has been observed for childhood cat keeping and adult cat acquisition in relation to asthma in a previous ECRHS publication by Svanes and colleagues [42]. For questionnaire based predictors, having the bed in the living room has been most consistently associated with adverse effects on respiratory health. According to the data, having the bed in the living room was not significantly associated with measured microbial compounds and there was further no association with questionnaire based signs of mould and dampness at home (*χ*^*2*^-test, data not shown). Nevertheless, having the bed in the living-room might stand for certain living-conditions or life-style factors which in turn might influence respiratory health status itself. Unfortunately, we are not able to investigate this possible interaction in more detail. Lastly, although there are several methods existing for dust collection on different sampling sites within the home, a publication by Wickens and colleagues recommended mattress dust as a representative sampling site for the measurement of allergens and endotoxin in the home environment [43]. Due to the cross-sectional nature of this investigation, reverse causation cannot be ruled out completely, but considered as reduced, at least for inspector observed exposure of mould and dampness.

## Conclusion

In conclusion, this investigation in a large adult population among 10 European countries provided information on the distribution and geographical variation of a range of fungal and bacterial agents measured with molecular and chemical methods in mattress dust. Indoor and personal characteristics such as signs of dampness or pet ownership could be identified as significant determinants for microbial agent concentrations. In particular, this study pointed out the impact of measured bacterial components in relation to respiratory symptoms. Muramic acid was associated with water damage at home, and was in turn also positively associated with current asthma symptoms.

## Additional file

Additional file 1:
**Detailed information about the selected home characteristics as possible predictors for microbial exposure in mattress dust.**

## Abbreviations

ECRHS: (European Community Respiratory Health Survey)
LPS: (Lipopolysaccharide) qPCR, (quantitative Polymerase Chain Reaction)
HITEA: (Health Effects of Indoor Pollutants: Integrating Microbial Toxicological and Epidemiological Approaches)
SOB: (Shortness of Breath)
FEV1: (Forced expiratory volume in 1 second) FVC, (Forced vital capacity)
IgE: (Immunoglobulin E)
IQR: (Interquartile range)
aMR 95%CI: (adjusted Mean Ratio 95% Confidence Interval)
MAMPs: Microorganism-associated molecular patterns
