# Supplementary material for: Predictors of microbial agents in dust and respiratory health in the Ecrhs
Source: BMC Pulm Med. 2015 May 2;15:48. doi: 10.1186/s12890-015-0042-y (PMC4425915; doi:10.1186/s12890-015-0042-y)
Supplement: Additional file 1: — Detailed information about the selected home characteristics as possible predictors for microbial exposure in mattress dust. [file 12890_2015_42_MOESM1_ESM.doc]

**Online supplement**: Detailed information about the selected home characteristics as possible predictors for microbial exposure in mattress dust

| **Indoor questionnaire / Indoor inspection** | **Original question:** |
| --- | --- |
| **MOULD/DAMPNESS** | Observed during inspection:  “*Are there any damp patches on the walls or ceilings in the in the bedroom/living room?”* |
|  | Observed during inspection:  *“Is there any mould or mildew on the walls or ceiling in the bedroom/living room?”* |
|  | Reported during inspection:  “*Do you get condensation on your bedroom/living room windows especially in the morning in the winter?”* |
|  |  |
| **Main questionnaire  (self-reported)** |  |
| **MOULD/DAMPNESS** | *“Has there been any water damage to the building or its contents, for example from broken pipes, leaks or floods?”* |
|  | *“Has there been any water damage in the last 12 months?”* |
|  | *“Within the last 12 months, have you had wet or damp spots on surfaces inside your home other than in the basement (for example on walls, wall paper, ceilings or carpets?)”* |
|  | *“Has there ever been any mould or mildew on any surface, other than food, inside the home?”* |
|  | *“Has there been mould or mildew on any surfaces inside the home in the last 12 months?”* |
| **CAT/DOG** | *“Do you keep a dog/cat?”* |
|  | *“Is your dog/cat (dogs/cats) allowed inside the house?”* |
|  | *“Is your dog/cat (dogs/cats) allowed in the bedroom?”* |
| **SMOKING** | *“Have you ever smoked for as long as a year?”* |
|  | *“Do you now smoke, as of one month ago?”* |
